# The perceived impact of location privacy: A web-based survey of public health perspectives and requirements in the UK and Canada

**DOI:** 10.1186/1471-2458-8-156

**Published:** 2008-05-09

**Authors:** Philip AbdelMalik, Maged N Kamel Boulos, Ray Jones

**Affiliations:** 1 Faculty of Health and Social Work, University of Plymouth, Centre Court, 73 Exeter Street, Drake Circus, Plymouth, Devon PL4 8AA, UK; 2 Office of Public Health Practice, Public Health Agency of Canada, 120 Colonnade Road, AL6702A, Ottawa, Ontario, K1A 0K9, Canada

## Abstract

**Background:**

The "place-consciousness" of public health professionals is on the rise as spatial analyses and Geographic Information Systems (GIS) are rapidly becoming key components of their toolbox. However, "place" is most useful at its most precise, granular scale – which increases identification risks, thereby clashing with privacy issues. This paper describes the views and requirements of public health professionals in Canada and the UK on privacy issues and spatial data, as collected through a web-based survey.

**Methods:**

Perceptions on the impact of privacy were collected through a web-based survey administered between November 2006 and January 2007. The survey targeted government, non-government and academic GIS labs and research groups involved in public health, as well as public health units (Canada), ministries, and observatories (UK). Potential participants were invited to participate through personally addressed, standardised emails.

**Results:**

Of 112 invitees in Canada and 75 in the UK, 66 and 28 participated in the survey, respectively. The completion proportion for Canada was 91%, and 86% for the UK. No response differences were observed between the two countries. Ninety three percent of participants indicated a requirement for personally identifiable data (PID) in their public health activities, including geographic information. Privacy was identified as an obstacle to public health practice by 71% of respondents. The overall self-rated median score for knowledge of privacy legislation and policies was 7 out of 10. Those who rated their knowledge of privacy as high (at the median or above) also rated it significantly more severe as an obstacle to research (*P *< 0.001). The most critical cause cited by participants in both countries was bureaucracy.

**Conclusion:**

The clash between PID requirements – including granular geography – and limitations imposed by privacy and its associated bureaucracy require immediate attention and solutions, particularly given the increasing utilisation of GIS in public health. Solutions include harmonization of privacy legislation with public health requirements, bureaucratic simplification, increased multidisciplinary discourse, education, and development of toolsets, algorithms and guidelines for using and reporting on disaggregate data.

## Background

Although "place" has been coined one of the three pillars of epidemiological data, only relatively recently has it garnered significant attention in the public health field, as Geographic Information Systems (GIS) have increasingly become more affordable, accessible, and intuitive. Indeed, the public health community's "place-consciousness" is on the rise as spatial analyses and GIS, now defined as part of the medical and health literature [1-3], are rapidly becoming key components of the public health professional's toolbox [4].

Privacy, an evolving "principle as old as the common law" [5], has been cited as an issue in a variety of public health events, reports, and media releases [6-11]. So much so, in fact, that one sometimes cannot help but wonder if privacy is, indeed, the enemy of public health [12], and whether they could ever peacefully co-exist [13]. A distinction should here be made between the related concepts of *privacy, confidentiality*, and *security *within the context of the current discussion. *Privacy *is attributable to the individual about whom identifiable information pertains, and refers to that individual's right to control such information, thereby freeing the individual from un-invited intrusion and identification. *Confidentiality *obligates others who have been entrusted with such information to respect the individual's privacy, and is therefore attributable to third parties; a breach of confidentiality violates the privacy of the individual because the individual has had no control over the release of the data. Finally, *security *refers to tools and methods used to safeguard confidentiality and privacy [14,15]. This research deals specifically with privacy issues as regulated and defined by legislation and ethical guidelines surrounding consent. From within this context, an individual's privacy is not deemed to have been violated if data shared in the absence of consent cannot be used to identify the individual. Exception clauses generally exist in legislation, allowing authorities to release personally identifiable data under various circumstances – such as where it is deemed to be in the best interest of society or where it is impractical to obtain consent. Examples include Section 60 of the UK's *Health and Social Care Act 2001 *[16], and Sections 8 and 7 of Canada's Privacy Act [17] and Personal Information Protection and Electronic Documents Act [18], respectively. While an analysis of privacy legislation as it pertains to health data and the concept of "place" is beyond the scope of this paper, suffice it to say that such clauses are often ambiguous and subjective, particularly when combined with vague definitions of "sensitive personal information" and the scale at which geographic data becomes "identifiable". The concept of *place*, for example, is not explicitly specified as "sensitive personal data" in the UK's *Data Protection Act 1988 *[19], nor in the generic *EU Data Protection Directive *of 1995 [20] (though it is explicitly mentioned in various telecommunications directives), but postcodes are specifically mentioned in a 2005 NHS data protection and medical research POSTnote [21]. In Canada's *Privacy Act *[17], "address" is specifically listed as "personal information", while in the *Personal Information Protection and Electronic Documents Act *[18], it is not (though implied). Such ambiguities deter the sharing of data, causing organisations and authorities to err on the side of caution and not release identifying information [22], including spatial data.

It is no surprise, therefore, that the increasing popularity of "place" in public health has further exacerbated the public health research-privacy debate. Traditional health-data anonymisation techniques, such as pseudonymisation and aggregation, cannot be applied to spatial data without significantly altering or destroying the spatial relationships under investigation [23-26], and hence the very reason for which they are to be used in the first place. The problem with "place" is that it is most useful at its most precise, granular scale [15,23]. Yet with increasing spatial precision and accuracy comes a corresponding increase in the risk of identification, and therefore a breach of privacy [15]. This becomes particularly troublesome when the spatial data is linked to health, social or demographic data. The development of methods by which to mitigate these risks continues to be an active area of research, but thus far, proposed solutions have limitations, risks and tradeoffs, and lack guidelines on their appropriate use. Consequently, the acquisition of geographic data tends to be either limited, or at a sub-optimal or unusable scale. Not only do privacy issues impact data acquisition and use for analysis, but also visualisation and dissemination of the results. Researchers have been able to "reverse engineer" maps, for example, to successfully re-identify individuals [27-29].

While the debate between the fields of privacy and public health has raged on for decades [5] despite their interdependence on one another [14], tension continues to rise in concert with the rampant growth of information technology and e-Health. From a health research perspective, both Canada and the UK place strong emphasis on evidence-based public health policies and services [6], yet in both countries, this seems to be hampered by privacy issues. While some argue that this debate is the product of a lack of understanding of the legislation and regulations by the public health community [14,30,31], there is little in the way of formal collection and synthesis of the corresponding views and perspectives of those directly involved in public health activities. This paper describes the views and requirements of public health professionals in Canada and the UK on privacy issues and spatial data, as collected through a web-based survey. Given that Canada's health care and public health systems were both largely modeled after those of the UK [6,32,33], that each continues to be studied by the other for improvements and lessons learned [6,34], and that privacy issues for public health have been cited in both, it is expected that survey responses in the two countries will also be similar.

## Methods

### Development & Content

The survey was first developed on paper in the summer of 2006, and piloted with select public health individuals in Canada and the UK. It was then submitted for privacy assessment by the Access to Information and Privacy Branch of Health Canada, and for ethics review and approval from the Health Canada Research Ethics Board and the Southwest Multicentre Research Ethics Committee in the UK. Throughout the process it was clear that the survey would be developed as a closed web-based survey, running between November 2006 and January 2007. The final paper versions of the survey are provided (see Additional files 1, 2, 3) and can also be found on the research website [35].

The paper survey was then converted to a web-version by the ALPHA Project [36] team at the Public Health Agency of Canada (PHAC), and piloted by the author and several colleagues within the PHAC. The survey launch was delayed by two weeks, with only some of the concerns identified during the pilot being implemented due to limitations of the ALPHA architecture. Issues and limitations with the design of the web-based survey are addressed in a later section.

Three versions of the survey were developed and launched: Canada-English, Canada-French and UK-English. A summary of the survey's structure and contents is given in Table 1.

**Table 1:** Sections of the survey

**Section**	**Title**	**Description**
I	A little about you...	Participant scope, roles, use of GIS, etc
II	Current access to data	Asks participants with current access to PID to score 15 kinds of PID* on various dimensions, such as ease and frequency of access, usefulness and importance, etc.
III	No current access to data	Asks participants without current access to PID to score same as above
IV	Privacy issues	Collects participant opinions on the overall impact of restricted access to PID on public health practice (research, surveillance, health service delivery, etc)
V	Current data holdings and provision to others...	Collects information on the sharing of PID within and between participant organisations
VI	Solutions and research	Presents two distinct solutions to overcome barriers posed by privacy to public health research, and gather participant views on usefulness, usability and preference for each
VII	Qualitative component	Allows participants to provide views and opinions on knowledge of privacy and confidentiality issues/legislation, impact of privacy, proposed research and solutions, and additional thoughts or comments
VIII	Further participation and contact	Allows participants to provide contact information if they choose, for follow-up, updates, or piloting of potential solution(s)

* For all participants: first name; last name; initials; sex; date of birth; date of death; registered GP or family physician; street address; postal code; community name; city/town/village; region/geographic area; latitude/longitude.

For Canadian participants: provincial health insurance plan number; hospital ID.

For UK participants: old NHS number; new NHS number

### Target

The survey targeted government, non-government and academic GIS labs and research groups involved in public health, as well as public health units (Canada), ministries, and observatories (UK). Potential participants were identified through web searches of public health sites, mailing databases, personal contact, referrals/word of mouth, and postings on the research website [35], a PHAC Public Health Portal website [37], and the NHS Public Health Informatics Community website [38].

### Participation

Potential participants were invited to participate through a standardised but personally addressed email outlining the reason for the invite, the mechanisms by which their contact information was retrieved, a brief summary of the research and survey, a description of the data handling methods, an estimate of the time it would take to complete the survey (approximately 20 minutes), a unique user ID and password, the URL to the survey site, the URL to the research website, and the principle investigator's contact information.

The survey website had no other content. In order to participate, invitees were required to (1) successfully log in, and (2) consent to participation. Only the most recent responses for any given user ID were collected, ensuring only one survey was completed per participant. The consent screen outlined the voluntary and anonymous nature of the survey, indicated the approximate time it would take to complete the survey, the risks and benefits to the participants, the intellectual property and ownership of all data collected, and the protection of any personal data provided under Canadian and UK law. Failure to successfully complete either of these two requirements resulted in termination of the survey. After consenting, participants were given the option to select their country and language of choice, and the relevant survey then commenced.

All questions included a "Skip" option. Progress through the survey required the selection of a response for each question, and participants could terminate the survey at any time or complete it over multiple sessions, at their convenience. Questions were not randomized or alternated, but adaptive questioning was utilized. Question types varied, and included single-choice, multiple-choice, scale, and free-form response questions, thereby collecting both quantitative and qualitative responses. There was typically only one question per screen with multiple potential responses, the maximum number of which was 17. Depending on the responses of the participants, the survey was distributed over approximately 40 screens.

Key questions addressed by the survey included the following:

- Is there a requirement for personally identifiable data, including spatial data?

- What spatial resolution is ideal for public health research?

- Is privacy perceived to be a significant obstacle to public health practice?

- How knowledgeable do public health professionals consider themselves on privacy?

- What is the most critical obstacle to the access and use of personally identifiable data?

- What are the views of the public health community on public awareness and perceptions?

- Which is preferred: raw, case-level data, or aggregated, anonymised data?

Collected responses were analysed using basic descriptive statistics and non-parametric methods in SAS 9.2. The Checklist for Reporting Results of Internet E-Surveys (CHERRIES) [39] was used as a guideline in the reporting of the web-based survey methodology.

## Results

Of 112 invitees in Canada and 75 in the UK, 66 (59%) and 28 (37%) participated in the survey, respectively. Of the Canadian participants, three responded to the French version. The completion proportion for Canada was 91%, and 86% for the UK.

There were no differences in the distribution of roles reported by participants in both countries, with most participants (49% in Canada; 64% in the UK) identifying their main role as falling within the research and analysis domain (Table 2). Participant expertise varied, and included aboriginal health (Canada only), chronic diseases, paediatric public health, infectious diseases, dental public health, emergency preparedness and response, environmental public health, ethics and public health law, food and nutrition, health services, injuries and disabilities, mental health and substance misuse, social determinants of health, surveillance, and education.

**Table 2:** Number and percent of survey participants by main role and geographical scope

	**Main Role**					
	
**Scope**	**Strategic decision/policy maker**	**Manager/Coordinator**	**Consultant**	**Research & Analysis**	**Front-Line Responder/Patient Care/Clinical**	**Other**
**Canadian Participants**						

North American or National	3 (4.5%)	6 (9%)	-	9 (13.6%)	1 (1.5%)	2 (3.0%)
Provincial/Territorial	1 (1.5%)	3 (4.5%)	4 (6.1%)	6 (9.1%)	-	2 (3.0%)
Local/Regional	2 (3.0%)	7 (10.6%)	1 (1.5%)	17 (25.8%)	1 (1.5%)	1 (1.5%)

**Totals**	**6 (9.1%)**	**16 (24.2%)**	**5 (7.6%)**	**32 (48.5%)**	**2 (3.0%)**	**5 (7.6%)**

**UK Participants**						

European or National	1 (3.6%)	1 (3.6%)	-	1 (3.6%)	-	-
Regional	2 (7.1%)	1 (3.6%)	2 (7.1%)	12 (42.9%)	-	-
Local	2 (7.1%)	-	-	4 (14.3%)	-	1 (3.6%)

**Totals**	**5 (17.9%)**	**2 (7.1%)**	**2 (7.1%)**	**17 (60.7%)**	**0 (0.0%)**	**1 (3.6%)**

*One UK participant who identified a main role in research and analysis declined a response to the question on scope.

No response differences were observed between the two countries on each of the key questions, and the overall, combined results are therefore reported. A summary of the findings is given in Table 3.

**Table 3:** Summary of findings

**Question**	**Response Summary**^†^	
1. Is there a requirement for personally identifiable data?	Yes	(93%)
2. What spatial resolution is ideal for public health research?	Lat/Long or address	(69%)
3. Is privacy perceived to be a significant obstacle to public health practice?	Yes	(71%)
4. How knowledgeable do public health professionals consider themselves on privacy?	High Knowledge*	(53%)
5. What is the most critical obstacle to the access and use of personally identifiable data?	Bureaucracy Legislation	(33%) (25%)
6. What are the views of the public health community on public awareness and perceptions?	Less than 30% of the public is aware	(84%)
7. Which is preferred: raw, case level data, or aggregated, anonymised data?	Raw, case-level data	(66%)

^†^Numbers in parentheses are the percent of participants who responded as described

*Participants rating their knowledge as high were also more likely to rate privacy as a more severe obstacle (*P *< 0.001)

### Is there a requirement for personally identifiable data, including spatial data?

Almost all participants identified a need for personally identifiable data (PID) in their roles; only one Canadian participant indicated no need for PID. Five Canadian participants and one UK participant chose not to answer the question. In total 93% of participants indicated a requirement for PID in their public health activities.

### What spatial resolution is ideal for public health research?

All participants identified geographic location of health data as a requirement for their roles or organisation. When asked "...what level of geography would you ideally like to visualise your data and/or conduct spatial analyses," 69% of respondents identified "latitude and longitude, exact street address, or exact household."

### Is privacy perceived to be a significant obstacle to public health practice? AND How knowledgeable do public health professionals consider themselves on privacy?

When asked "Are you or have you been restricted in your use of GIS for any public health activity because of privacy concerns (i.e. map or data might identify an individual or community)?" 79% of respondents marked "YES".

Of 83 participants who responded to the question "In your opinion, do current restrictions to PID pose an obstacle to any aspects of public health practice?" 59 (71%) agreed, rating the obstacle severity at 6 or higher. Of these 59, 36 (61%) rated their knowledge of privacy and confidentiality issues/legislation at 6 out of 10 or higher, with a mean score of 7.5 (std = 1.0) and a median score of 7.

Using the median, respondents with a self-rated knowledge score lower than 7 were classified as "low" on knowledge (47%), while those at or above the median score were classified as "high" (53%). Those classified as high were more likely to rate privacy as an obstacle (one-sided Wilcoxon exact *P *< 0.001). A trend was evident for the overall correlation between restriction score and self-rated privacy knowledge score (Spearman *r *= 0.22, *P *= 0.057).

### What is the most critical obstacle to the access and use of personally identifiable data?

The most common obstacles were reported as bureaucracy and legislation by 33% and 25% of the participants, respectively. Other responses included public disapproval/paranoia (15%), practitioner paranoia (7%), lack of knowledge (6%), combination of these factors (4%), other (2%), and none (skipped question, 7%).

### What are the views of the public health community on public awareness and perceptions?

Fifty seven percent of participants felt that under 10% of the public population is aware of the impact of restricted access to PID on public health practice; 74% felt it to be under 20%, and 84% felt the proportion to be less than 30% (cumulative frequencies). Most identified education and awareness (through media, reports, case studies, scenarios, etc) as the best methods to increase this proportion. When then asked what proportion of the public they felt would allow the use of their PID if they were educated on the usefulness of such data to public health practice, 67% said 50% or higher.

### Which is preferred: raw, case-level data, or aggregated, anonymised data?

More respondents identified a preference for having access to granular-level rather than aggregate data (53 vs. 27; 66% of those responding to this question).

## Discussion

This survey and user-needs assessment on privacy and public health shows a definite requirement by public health professionals – in various fields and positions in both Canada and the UK – for personally identifiable data, including spatial data. The requirement for this spatial data is at its most granular level – latitude and longitude, or exact street address – which necessarily compromises patient privacy. It is not surprising, therefore, that public health professionals perceive privacy to be a significant obstacle to public health practice.

There are those who would argue that this perception is the product of a lack of understanding of the legislation and regulations by the public health community. The results of this research, however, indicate the contrary. Not only did public health professionals in both countries generally rate themselves high on knowledge of privacy legislation and related issues, but those with the highest self-rated scores also tended to rate privacy as more of an obstacle. That these self-ratings of knowledge are not representative of actual knowledge remains possible.

Participants perceived the most critical obstacles to sharing or acquisition of health data with PID to be bureaucracy, followed by legislation.

Bureaucracy surrounding health research in both Canada and the UK generally revolves around data ownership, academic competitiveness, ethics review boards or committees, and in particular, requirements for informed consent, even if they compromise public health, or are not in the best interests of the patients involved [40-42]. Since seeking subject consent with every new hypothesis to be tested or model to be developed is an impossible task, some have suggested that thought be given to "blanket" consent. At the Canadian Institutes for Health Research (CIHI) 2003 workshop on the legal and ethical issues facing the Canadian Lifelong Health Initiative [43], participants spent some time discussing such issues, only to emphasise the importance of the establishment of ethical governance and structure; essentially, more necessary bureaucracy. Interestingly, while the debate continues, a relatively recent survey found that most of the British public did not consider the use of their National Cancer Registry PID for public health research and surveillance to be an invasion of their privacy [30]. While the ethics of blanket consent are not discussed in this study, it is nonetheless offered as a potential solution in light of the requirements of the public health community. This does not, however, address other issues of data ownership and control that contribute to the bureaucratic debate.

While many individuals recognised the importance of privacy legislation, participants generally indicated a concern and, in some cases, first-hand frustration that legislation unduly restricts public health activities, compromising surveillance and research. Many phrases were used by respondents to describe the implications of privacy legislation on public health, including, among others: "increasingly restrictive;" "serious;" "incomplete;" "fuzzy;" "does more harm than good;" "two-edged sword;" "causes challenges;" "delays and restricts access [to data];" " [is a] hindrance to the improvement and efficiency of public health;" "disappointing;" "frustrating;" "difficult to interpret;" "very worrisome;" "disadvantages the public interest;" "not properly understood;" "over-protective;" "limiting;" "hinders knowledge;" and "used as an excuse not to share data." A large proportion of the public health community represented in this sample clearly expressed major concerns with the impact of privacy legislation on their work – both in Canada, and in the UK – in spite of having a good understanding and acceptance of its purpose and necessity. It is also important for legislation to be written in an unambiguous manner that is clearly understood by both public health professionals and the general public [4].

Public health professionals are largely of the opinion that the general public's level of awareness of the impact of restricted access to PID on public health practice is extremely low. Surveys by the Office of the Privacy Commissioner in Canada [44] repeatedly show that the majority of Canadians surveyed (up to 80%) place an extremely high level of importance on strong laws to protect personal information, particularly health information, and that they feel that the level of protection of their personal information has declined over the past ten years. Yet interestingly, only 20% are clearly aware of existing laws, and even fewer (12%) are aware of their rights around the collection, use and disclosure of this information. The "need to raise Canadians' awareness about the current laws in place and what their rights are" [44] must therefore be coupled with the corresponding need to address this from within the context of public health requirements.

Educating the public, therefore, as well as practitioners, data users, policy makers and politicians, was not surprisingly identified by participants as a potential solution. Participants put emphasis on the utilisation of the media to educate and increase awareness, as well as demonstrating the impact of a lack of data, and the benefits of its use when available. Demonstration of the benefits to the individual (e.g. streamlining of the system, not being asked for personal information with every visit to a new clinician, improved dissemination of public health information and intelligence directly to the public) was also offered as a solution, and summed up by one participant in the phrase "seeing is believing". It is worth noting, however, that a number of participants displayed a certain level of pessimism that until a crisis or extreme event occurs, no amount of education or awareness-increasing activities would make a difference.

Public health professionals generally prefer disaggregate, case-level data, but access to this data is an issue. The limitations imposed by privacy on public health have resulted in the development of a variety of techniques for data anonymisation [15,23,45]. However, all unavoidably have their issues, risks and limitations, and there is currently no framework to guide public health professionals in their appropriate use and interpretation.

### Generalisability

Although the findings of this paper may be generalisable to public health professionals in Canada and the UK, issues of privacy and public health are not unique to these countries. Privacy is defined as a fundamental human right in the legislation of many countries, and the concept is enshrined in Article 12 of the United Nations' *Universal Declaration of Human Rights *[46] and Article 8 of the *European Convention on Human Rights *[47]. Similarly, public health is an international discipline; both diseases and information are ubiquitous, and neither is constrained by political boundaries and oceans. The increasing requirement for spatial data and its inherent clash with privacy legislation therefore extend beyond the UK and Canadian contexts, and the results, requirements and conclusions drawn from this research can be generalised to wherever such a clash exists. The implementation of solutions by national governments may be further exacerbated by issues of social political trust. General public distrust in government initiatives and motives, such as in most countries of the European Union, Canada, and the United States [48,49], complicates changes that may be perceived by the public to be intrusions of privacy. Such issues may currently be less of a concern in countries such as Finland, Sweden, Denmark, and the Netherlands, where social political trust, though declining, has traditionally tended to be much higher [50-53]. However, even in such nations where privacy and health have traditionally not clashed, increased international data sharing requirements and spatial data implications may pose unanticipated and challenging obstacles.

### Limitations

No comprehensive lists of public health and health GIS professionals were found in either country, so it was not possible to invite a random sample. In addition, the response rate in the UK was relatively low, and it is therefore uncertain that the sample is representative of all public health professionals in the two countries. However, responses between the two countries were consistent, with no significant differences.

Since knowledge of privacy legislation and policies was based on self-rated scores, a thorough review and assessment of privacy legislation as it pertains to public health practice is required in both Canada and the UK to validate the findings of this survey.

A number of limitations and issues pertaining to the web-survey were identified. Most notable of these was the presence of a scroll bar in sections II and III which most participants missed, thereby eliminating the ability to capture items in reference to "place", such as usefulness. However, these items were also captured more broadly in other sections of the survey. Other issues involved the inability of the architecture to support various designs and types of questions that would have facilitated the completion of the survey, and shortened the length of time required. Participants also noted frustration with the navigation and structure of the survey pages. A document outlining these issues and others was submitted to the ALPHA team after the initial pilot for future enhancements to the architecture.

## Conclusion

It is clear that privacy is perceived to be a major obstacle and issue for public health – the literature illustrates it, and the current study provides both quantitative and qualitative evidence. Together, these provide a more holistic portrayal of public health community viewpoints, and can be used to educate the public, and as evidence for decision makers to implement changes in policies and legislation. The clash between a requirement for personally identifiable data – including exact, individual location – by public health professionals, and the limitations imposed by privacy and its associated bureaucracy, must be addressed and appropriate solutions developed, particularly given the increasing utilisation of geographic information systems in public health and the imminent completion of comprehensive electronic health systems. Privacy legislation is critical for the protection of this fundamental human right, and to prevent the abuse of personal information, particularly in the health field. However, the legislation must be harmonised with the requirements of public health practice if the health of societies and populations is to be maintained and improved. Since health is not limited by political boundaries, this must be pursued at an international level, and solutions must address these perceptions in the public health community, simplify the bureaucratic process, promote multidisciplinary discussions between legislators, bureaucrats and the public health community, educate communities, and develop and provide public health professionals with toolsets, algorithms and guidelines for using and reporting on disaggregate data. While the results of this study should inform and justify the development of techniques that better anonymise health data with minimal impact on its integrity and frameworks for implementing them, it seems fitting to echo the warning of Curtis et al: "...health and spatial scientists should be proactive and suggest a series of point level spatial confidentiality guidelines before governmental decisions are made which may be reactionary toward the threat of revealing confidential information, thereby imposing draconian limits on research using a GIS [27]."

## Competing interests

PA is an epidemiologist working directly with Geographic Information Systems, and pursuing PhD research on issues of privacy and spatial health information. There are no other competing interests.

## Authors' contributions

PA conceived, designed and carried out the survey (part of his PhD research at the Peninsula Postgraduate Health Institute, Plymouth, UK), and analysed the results. He also conducted the literature review and wrote the draft paper. MNKB and RJ reviewed and provided critical input to the study design and to the discussion and interpretation of its results and their implications. They also helped with the ethical approval process in the UK, identifying UK respondents, the literature review, and the revision of the draft paper. All authors have read and approved the final manuscript.

## Pre-publication history

The pre-publication history for this paper can be accessed here:



## Supplementary Material

Additional file 1 The impact of privacy on public health practice: Public health professional questionnaire, CANADA. This is the paper version of the Canadian-English survey that was adapted for the web to collect public health professional perspectives and requirements on location-privacy in Canada. Click here for file

Additional file 2 L'impact de la protection des renseignements personnels sur la pratique en santé publique, CANADA. This is the paper version of the Canadian-French survey that was adapted for the web to collect public health professional perspectives and requirements on location-privacy in Canada. Click here for file

Additional file 3 The impact of privacy on public health practice: Public health professional questionnaire, UNITED KINGDOM. This is the paper version of the UK survey that was adapted for the web to collect public health professional perspectives and requirements on location-privacy in Canada. Click here for file
