# Supplementary material for: The perceived impact of location privacy: A web-based survey of public health perspectives and requirements in the UK and Canada
Source: BMC Public Health. 2008 May 9;8:156. doi: 10.1186/1471-2458-8-156 (PMC2396622; doi:10.1186/1471-2458-8-156)
Supplement: Additional file 1 — The impact of privacy on public health practice: Public health professional questionnaire, CANADA. This is the paper version of the Canadian-English survey that was adapted for the web to collect public health professional perspectives and requirements on location-privacy in Canada. [file 1471-2458-8-156-S1.pdf]

The Impact of Privacy on Public Health Practice
Public Health Professional Questionnaire
CANADA

ESTIMATED TIME TO COMPLETE THIS QUESTIONNAIRE IS 20 TO 30 MINUTES

Thank you for taking the time to complete this questionnaire – your contribution is highly valued and critical to the research.

This questionnaire is divided into eight short sections to better categorize and organize the type of information being captured. You may skip any question(s) you would rather not answer, however please keep in mind that this compromises the quality of the research. Please also note that, unless you choose to complete section VIII of the questionnaire, your responses will remain anonymous and cannot be linked back to you. Therefore, please ensure that all responses are clearly marked.

Please note that your responses will vest in, and remain the property of Her Majesty the Queen in Right of Canada, and as such, may also be made available to and used by the Public Health Agency of Canada to improve their operations and service provision to the public health community. As an Office of Public Health Practice, Public Health Agency of Canada survey, all data collected, including your personal information, will be protected according to the Access to Information Act and the Privacy Act in Canada.

SECTION I – A little about you… (~ 5 minutes)

I-1 What would you say is the scope of the **bulk** of your involvement in public health?
(If more than one, please select only your **main** involvement)

☐ International ☐ N. American ☐ National ☐ Provincial/ Territorial ☐ Regional/ Local

I-2 With which public health organization are you currently employed / affiliated?
(If more than one, please select only your **main** organization)

- ☐ Public Health Agency of Canada
- ☐ Health Canada
- ☐ Other Federal Government Agency
- ☐ Provincial Government (e.g. Ministry of Health)
- ☐ Regional / Local Health Authority or Unit
- ☐ Canadian Public Health Association
- ☐ Other Non-Government Association (e.g. provincial epidemiological, diabetes, cancer, etc)
- ☐ University / Acadaemia
- ☐ Other
  Please specify: ____

I-3. Please indicate your current specific area(s) of expertise: (Check as many as apply)

- ☐ Aboriginal Health
- ☐ Chronic Diseases (cancer, diabetes, etc)
- ☐ Child / Paediatric Public Health
- ☐ Communicable / Infectious Diseases
- ☐ Dental Public Health
- ☐ Emergency Preparedness & Response
- ☐ Environment (pollution, climate, water & food safety, etc)
- ☐ Ethics, Public Health Law, Privacy, etc
- ☐ Other
- ☐ Food & Nutrition
- ☐ Genetics
- ☐ Health Services (needs, delivery, etc)
- ☐ Injuries / Disability
- ☐ Mental Health & Substance Misuse
- ☐ Occupational Health
- ☐ Social Determinants of Health (e.g. poverty, education, social exclusion, etc)
- ☐ Surveillance

  Please specify: ____

I-4. Which of the following **best** describe your roles or functions as a public health professional? (If more than one, please select only your **main** roles)

- ☐ Strategic decision / policy maker
- ☐ Manager or Coordinator
- ☐ Consultant
- ☐ Other
- ☐ Research and Analysis
- ☐ Front-line responder / patient care / clinical

  Please specify: ____

I-5. Thinking of your regular activities, how much of your time (roughly, as a percentage) would you typically spend doing each of the following?

Strategic decision / policy making	______________%
Management / Coordination	______________%
Consultation	______________%
Research / Analysis	______________%
Front-line response / patient care / clinical	______________%
Other (as specified in I-4)	______________%

I-6. In which of the roles you identified above are you **most likely** to use or require the use of personally identifiable data?

- ☐ Strategic decision / policy maker
- ☐ Manager or Coordinator
- ☐ Consultant
- ☐ Other
  Please specify: ____
- ☐ Research and Analysis
- ☐ Front-line responder / patient care / clinical

I-7 Do you have or foresee a need for including geographic location of health data in your roles or organization?

☐ YES ☐ NO

I-8 Geographic Information Systems (GIS) are tools that allow you to visualise and analyse your data spatially – that is, using their geographical location on earth. In which of the roles you identified above would GIS be useful?

- ☐ Strategic decision / policy maker
- ☐ Manager or Coordinator
- ☐ Consultant
- ☐ Other
  Please specify: ____
- ☐ Research and Analysis
- ☐ Front-line responder / patient care / clinical

I-9 What GIS application(s) do you currently use, or have you used in the past?

- ☐ Public Health Map Generator (PHMG)
- ☐ Other web-based: specify ________________________________

Desktop GIS products:

- ☐ ESRI ArcGIS products
- ☐ MapInfo
- ☐ AutoDesk products
- ☐ PCI Geomatics products
- ☐ Intergraph products
- ☐ Other
  Please specify: ____

- ☐ I have never used any GIS applications, and have no use for them
- ☐ I have never used any GIS applications, but am interested in learning more

I-10 At what level(s) of geography do you visualise your data and/or conduct spatial analyses for each product you use?

	PHMG	Other Web-Based	Desktop GIS
Latitude and Longitude	☐	☐	☐
Street address	☐	☐	☐
Dissemination area	☐	☐	☐
Postal Code	☐	☐	☐
Census Subdivision	☐	☐	☐
Census Division	☐	☐	☐
Forward Sortation Area	☐	☐	☐
Urban – Rural	☐	☐	☐
Provincial	☐	☐	☐
Other			

Please specify: ____

I-11 Are you or have you been restricted in your use of GIS for any public health activity because of privacy concerns (i.e. map or data might identify an individual or community)?

☐ YES → Please explain ☐ No → Please explain

I-12 Setting privacy issues aside and in light of your response to the previous question, at what level(s) of geography would you ***ideally*** like to visualise your data and/or conduct spatial analyses for each product you use?

	PHMG	Other Web-Based	Desktop GIS
Latitude and Longitude	☐	☐	☐
Street address	☐	☐	☐
Dissemination area	☐	☐	☐
Postal Code	☐	☐	☐
Urban – Rural	☐	☐	☐
Other			

Please specify: ____

SECTION II – Current access to data

(~ 4 minutes)

The questions in this section all pertain to the role you identified in question I-6 in Section I. If you do **not** have access to any of the *Personally Identifiable Data* (*PID*) listed in question II-1, please mark the last option in question II-1 and skip to Section III – No current access to data. Otherwise, please complete this section, and then skip to Section IV – Privacy Issues.

NOTE: **The term "access" as used in this survey implies the ability to actually *acquire* individual level data so you can work with it directly.**

II-1. What PID do you currently have access to? (Check as many as apply)

- ☐ First Name
- ☐ Last Name
- ☐ Initials
- ☐ Sex
- ☐ Date of Birth / Age
- ☐ Date of Death
- ☐ Provincial Health Insurance Plan Number
- ☐ Hospital ID
- ☐ Registered GP / Family Physician
- ☐ Street Address
- ☐ Postal Code
- ☐ Community Name
- ☐ City / Town / Village
- ☐ Region / Geographic Area
- ☐ Latitude & Longitude

- ☐ Other
  Please specify: ____

- ☐ **I do NOT currently have access to any of the above (please skip to Section III)**

The following questions all pertain to the *PID* you have access to, as identified in the previous question.
For all scales, circle '0' if you "Don't know"

II-2 From a privacy and organisational bureaucracy perspective, how easy would you say it is for you to access this *PID* when you need it?
Please circle the appropriate number, 1 being "Extremely difficult", and 10 being "Very easy"

Extremely Difficult → Very easy

	Don't Know	1	2	3	4	5	6	7	8	9	10
First Name	○	○	○	○	○	○	○	○	○	○	○
Last Name	○	○	○	○	○	○	○	○	○	○	○
Initials	○	○	○	○	○	○	○	○	○	○	○
Sex	○	○	○	○	○	○	○	○	○	○	○
Date of Birth / Age	○	○	○	○	○	○	○	○	○	○	○
Date of death	○	○	○	○	○	○	○	○	○	○	○
Provincial Health Insurance Plan Number	○	○	○	○	○	○	○	○	○	○	○
Hospital ID	○	○	○	○	○	○	○	○	○	○	○
Registered GP / Family Physician	○	○	○	○	○	○	○	○	○	○	○
Street Address	○	○	○	○	○	○	○	○	○	○	○
Postal Code	○	○	○	○	○	○	○	○	○	○	○
Community Name	○	○	○	○	○	○	○	○	○	○	○
City / Town / Village	○	○	○	○	○	○	○	○	○	○	○
Region / Geographic Area	○	○	○	○	○	○	○	○	○	○	○
Latitude / Longitude	○	○	○	○	○	○	○	○	○	○	○

II-3. On average, how often do you access the *PID* you identified above?

Rarely ——→ All the time

	Not Applicable	1	2	3	4	5	6	7	8	9	10
First Name	○	○	○	○	○	○	○	○	○	○	○
Last Name	○	○	○	○	○	○	○	○	○	○	○
Initials	○	○	○	○	○	○	○	○	○	○	○
Sex	○	○	○	○	○	○	○	○	○	○	○
Date of Birth / Age	○	○	○	○	○	○	○	○	○	○	○
Date of death	○	○	○	○	○	○	○	○	○	○	○
Provincial Health Insurance Plan Number	○	○	○	○	○	○	○	○	○	○	○
Hospital ID	○	○	○	○	○	○	○	○	○	○	○
Registered GP / Family Physician	○	○	○	○	○	○	○	○	○	○	○
Street Address	○	○	○	○	○	○	○	○	○	○	○
Postal Code	○	○	○	○	○	○	○	○	○	○	○
Community Name	○	○	○	○	○	○	○	○	○	○	○
City / Town / Village	○	○	○	○	○	○	○	○	○	○	○
Region / Geographic Area	○	○	○	○	○	○	○	○	○	○	○
Latitude / Longitude	○	○	○	○	○	○	○	○	○	○	○

II-4. How useful / important is this *PID* to you and your roles and responsibilities?
Please circle the appropriate number, with 1 being "Not at all useful", and 10 being "Critical to my roles and responsibilities"

Not at all Useful ——→ Critical

	Don't Know	1	2	3	4	5	6	7	8	9	10
First Name	○	○	○	○	○	○	○	○	○	○	○
Last Name	○	○	○	○	○	○	○	○	○	○	○
Initials	○	○	○	○	○	○	○	○	○	○	○
Sex	○	○	○	○	○	○	○	○	○	○	○
Date of Birth / Age	○	○	○	○	○	○	○	○	○	○	○
Date of death	○	○	○	○	○	○	○	○	○	○	○
Provincial Health Insurance Plan Number	○	○	○	○	○	○	○	○	○	○	○
Hospital ID	○	○	○	○	○	○	○	○	○	○	○
Registered GP / Family Physician	○	○	○	○	○	○	○	○	○	○	○
Street Address	○	○	○	○	○	○	○	○	○	○	○
Postal Code	○	○	○	○	○	○	○	○	○	○	○
Community Name	○	○	○	○	○	○	○	○	○	○	○
City / Town / Village	○	○	○	○	○	○	○	○	○	○	○
Region / Geographic Area	○	○	○	○	○	○	○	○	○	○	○
Latitude / Longitude	○	○	○	○	○	○	○	○	○	○	○

II-5. What impact would removal of your access to this *PID* have on the quality of your work and resulting public health decisions?
Please circle the appropriate number, with 1 being "No impact – quality would not suffer", and 10 being "Severe Impact - results and decisions would be severely compromised"

No Impact → Severe Impact

	Don't Know	1	2	3	4	5	6	7	8	9	10
First Name	○	○	○	○	○	○	○	○	○	○	○
Last Name	○	○	○	○	○	○	○	○	○	○	○
Initials	○	○	○	○	○	○	○	○	○	○	○
Sex	○	○	○	○	○	○	○	○	○	○	○
Date of Birth / Age	○	○	○	○	○	○	○	○	○	○	○
Date of death	○	○	○	○	○	○	○	○	○	○	○
Provincial Health Insurance Plan Number	○	○	○	○	○	○	○	○	○	○	○
Hospital ID	○	○	○	○	○	○	○	○	○	○	○
Registered GP / Family Physician	○	○	○	○	○	○	○	○	○	○	○
Street Address	○	○	○	○	○	○	○	○	○	○	○
Postal Code	○	○	○	○	○	○	○	○	○	○	○
Community Name	○	○	○	○	○	○	○	○	○	○	○
City / Town / Village	○	○	○	○	○	○	○	○	○	○	○
Region / Geographic Area	○	○	○	○	○	○	○	○	○	○	○
Latitude / Longitude	○	○	○	○	○	○	○	○	○	○	○

II-6. What *PID* do you currently **NOT** have access to, but believe would be beneficial to you to further enhance your work and resulting public health decisions? (Check as many as apply)

- ☐ First Name
- ☐ Last Name
- ☐ Initials
- ☐ Sex
- ☐ Date of Birth / Age
- ☐ Date of Death
- ☐ Provincial Health Insurance Plan Number
- ☐ Hospital ID
- ☐ Registered GP / Family Physician
- ☐ Street Address
- ☐ Postal Code
- ☐ Community Name
- ☐ City / Town / Village
- ☐ Region / Geographic Area
- ☐ Latitude & Longitude

- ☐ Other
  Please specify: ______________________________

- ☐ None

Please skip to Section IV – Privacy Issues

SECTION III – No current access to data

(~ 2 minutes)

If you have access to *Personally Identifiable Data* (*PID*) and completed Section II above, then please skip to Section IV – Privacy Issues.
For all scales, circle '0' if you "Don't know"

NOTE: **The term "access" as used in this survey implies the ability to actually *acquire* individual level data so you can work with it directly.**

III-1. Having access to which of the following *PID* would facilitate your roles and responsibilities, or enhance your work and improve resulting public health decisions? (Check as many as apply)

- ☐ First Name
- ☐ Last Name
- ☐ Initials
- ☐ Sex
- ☐ Date of Birth / Age
- ☐ Date of Death
- ☐ Provincial Health Insurance Plan Number
- ☐ Hospital ID
- ☐ Registered GP / Family Physician
- ☐ Street Address
- ☐ Postal Code
- ☐ Community Name
- ☐ City / Town / Village
- ☐ Region / Geographic Area
- ☐ Latitude & Longitude

- ☐ Other
  Please specify: ____

- ☐ None

III-2. How useful to you and your roles and responsibilities (as identified in Section I) would access to the *PID* you identified above be?
Please circle the appropriate number, with 1 being "Not at all useful", and 10 being "Very useful – would greatly enhance by roles and responsibilities"

Not at all Useful → Very Useful

	Don't Know	1	2	3	4	5	6	7	8	9	10
First Name	○	○	○	○	○	○	○	○	○	○	○
Last Name	○	○	○	○	○	○	○	○	○	○	○
Initials	○	○	○	○	○	○	○	○	○	○	○
Sex	○	○	○	○	○	○	○	○	○	○	○
Date of Birth / Age	○	○	○	○	○	○	○	○	○	○	○
Date of death	○	○	○	○	○	○	○	○	○	○	○
Provincial Health Insurance Plan Number	○	○	○	○	○	○	○	○	○	○	○
Hospital ID	○	○	○	○	○	○	○	○	○	○	○
Registered GP / Family Physician	○	○	○	○	○	○	○	○	○	○	○
Street Address	○	○	○	○	○	○	○	○	○	○	○
Postal Code	○	○	○	○	○	○	○	○	○	○	○
Community Name	○	○	○	○	○	○	○	○	○	○	○
City / Town / Village	○	○	○	○	○	○	○	○	○	○	○
Region / Geographic Area	○	○	○	○	○	○	○	○	○	○	○
Latitude / Longitude	○	○	○	○	○	○	○	○	○	○	○

III-3 How easy would it be for you to access the *PID* you identified above, if you were to need it?
Please circle the appropriate number, with 1 being “Impossible”, and 10 being “Very easy”

Impossible → Very Easy

	Don’t Know	1	2	3	4	5	6	7	8	9	10
First Name	○	○	○	○	○	○	○	○	○	○	○
Last Name	○	○	○	○	○	○	○	○	○	○	○
Initials	○	○	○	○	○	○	○	○	○	○	○
Sex	○	○	○	○	○	○	○	○	○	○	○
Date of Birth / Age	○	○	○	○	○	○	○	○	○	○	○
Date of death	○	○	○	○	○	○	○	○	○	○	○
Provincial Health Insurance Plan Number	○	○	○	○	○	○	○	○	○	○	○
Hospital ID	○	○	○	○	○	○	○	○	○	○	○
Registered GP / Family Physician	○	○	○	○	○	○	○	○	○	○	○
Street Address	○	○	○	○	○	○	○	○	○	○	○
Postal Code	○	○	○	○	○	○	○	○	○	○	○
Community Name	○	○	○	○	○	○	○	○	○	○	○
City / Town / Village	○	○	○	○	○	○	○	○	○	○	○
Region / Geographic Area	○	○	○	○	○	○	○	○	○	○	○
Latitude / Longitude	○	○	○	○	○	○	○	○	○	○	○

III-4. What impact has your lack of access to this *PID* had on the quality of your work and resulting public health decisions?
Please circle the appropriate number, with 1 being “No impact – quality has not suffered”, and 10 being “Severe Impact - results and decisions have been severely compromised”

No Impact → Severe Impact

	Don’t Know	1	2	3	4	5	6	7	8	9	10
First Name	○	○	○	○	○	○	○	○	○	○	○
Last Name	○	○	○	○	○	○	○	○	○	○	○
Initials	○	○	○	○	○	○	○	○	○	○	○
Sex	○	○	○	○	○	○	○	○	○	○	○
Date of Birth / Age	○	○	○	○	○	○	○	○	○	○	○
Date of death	○	○	○	○	○	○	○	○	○	○	○
Provincial Health Insurance Plan Number	○	○	○	○	○	○	○	○	○	○	○
Hospital ID	○	○	○	○	○	○	○	○	○	○	○
Registered GP / Family Physician	○	○	○	○	○	○	○	○	○	○	○
Street Address	○	○	○	○	○	○	○	○	○	○	○
Postal Code	○	○	○	○	○	○	○	○	○	○	○
Community Name	○	○	○	○	○	○	○	○	○	○	○
City / Town / Village	○	○	○	○	○	○	○	○	○	○	○
Region / Geographic Area	○	○	○	○	○	○	○	○	○	○	○
Latitude / Longitude	○	○	○	○	○	○	○	○	○	○	○

SECTION IV – Privacy Issues

(~ 3 minutes)

This section pertains to the field of public health in general, and uses the term "*public health practice*" to refer to its various activities, including research, surveillance, health service delivery, strategic policy and decision making, etc. The goal is to get your opinion, as a public health professional, on the overall impact of restricted access to *PID* on *public health practice* in Canada. These questions ask for your opinion; if you're not sure how to answer a question in this section, please just hazard a guess!

NOTE: **The term "access" as used in this survey implies the ability to actually *acquire* individual level data so you can work with it directly.**

IV-1. In your opinion, do current restrictions on access to *PID* pose an obstacle to any aspects of *public health practice* (e.g. research, surveillance, etc)?
Please circle the appropriate number, with 1 being "Not an obstacle at all", and 10 being "Yes, they pose a serious threat to accurate public health practice"

1	2	3	4	5	6	7	8	9	10

Not an issue → Serious Threat

IV-2. How amenable would ***you*** be to other professionals in the public health field having access to ***your*** *PID* for public health research and analyses (e.g. **your** address, date of birth, etc) to improve public health delivery, service, etc?

☐ Sure, go ahead ☐ No Way ☐ Not Sure → Please Explain

IV-3a. In your opinion, what proportion of the public is aware of the impact of restricted access to *PID* on public health practice? (Please just guess!)
Please circle the approximate proportion

10%	20%	30%	40%	50%	60%	70%	80%	90%	100%

No one → Everyone

IV-3b. How do you think we could increase this proportion?

IV-4a. In your opinion, what proportion of the public would allow the use of *PID* for public health practice if they were asked and educated on the usefulness of such data to public health practice? (Please just guess!) - Please circle the approximate proportion

10%	20%	30%	40%	50%	60%	70%	80%	90%	100%

No one → Everyone

IV-4b. How do you think we could increase this proportion?

SECTION V – Current data holdings and provision to others... (~ 2 minutes)

This section gathers information on the sharing of PID within and between organizations.
For all scales, circle '0' if you "Don't know"

V-1. What would you say is the one **most** critical obstacle in the sharing or acquisition of PID linked to health data? (Please select only one; give your opinion!)

☐ National legislation ☐ Public disapproval ☐ Organizational bureaucracy
☐ Lack of knowledge ☐ Public Paranoia ☐ Practitioner Paranoia

☐ Other (please specify): ____________________

V-2. Do you or your organization currently collect individual-level health data for any purpose (e.g. research, surveillance, service delivery, etc), or act as the custodian of such data?

☐ YES → **Continue** ☐ NO → **Go to Section VI**

V-3. For what specific purpose(s) is this data collected? (check as many as apply)

☐ Research ☐ Surveillance ☐ Service Delivery
☐ Other
Please specify: ____________________

V-4. What data is collected?

☐ First Name
☐ Last Name
☐ Initials
☐ Sex
☐ Date of Birth / Age
☐ Date of Death
☐ Provincial Health Insurance Plan Number
☐ Hospital ID
☐ Registered GP / Family Physician
☐ Street Address
☐ Postal Code
☐ Community Name
☐ City / Town / Village
☐ Region / Geographic Area
☐ Latitude & Longitude

☐ Other
Please specify: ____________________

V-5. How difficult is it for other public health professionals such as yourself to acquire access to your PID and linked health data holdings if they are outside your immediate working team, but within…

Impossible → Very Easy

	D/K	1	2	3	4	5	6	7	8	9	10
Your organisation?	○	○	○	○	○	○	○	○	○	○	○
The Federal Government?	○	○	○	○	○	○	○	○	○	○	○
Your Provincial Government?	○	○	○	○	○	○	○	○	○	○	○
A Provincial Government other than your own?	○	○	○	○	○	○	○	○	○	○	○
A regional or public health authority?	○	○	○	○	○	○	○	○	○	○	○
A University or Research Facility	○	○	○	○	○	○	○	○	○	○	○
Another national government (e.g. CDC in the US, NHS in the UK, etc)	○	○	○	○	○	○	○	○	○	○	○
The World Health Organization	○	○	○	○	○	○	○	○	○	○	○

SECTION VI – Solutions and Research

(~ 7 minutes)

The proposed research will seek to apply a method (called a *transformation*) to public health data such that important relationships within and between the data are preserved, but the actual identity of the individual is anonymized. So, for example, if you were looking at an infectious outbreak in children, you might be interested in where the infected children are relative to one another, as well as where the schools are, arenas, community centres, etc. You would then preserve the *relationship* between these points of interest, and change everything else, so that the original points can no longer be identified back to their original owners. In this way, you have *transformed* the data so that you're still looking at individual-level data, but can't determine who it belongs to (i.e., it has become anonymous). Assuming the data custodians allow the data derived from such a *transformation* to be made available to the public health professional community:
For all scales, circle '0' if you "Don't know"

VI-1. How useful would such a transformation be to you in your current role?
Please circle the appropriate number, with 1 being "Not at all useful", and 10 being "Very useful"

0	1	2	3	4	5	6	7	8	9	10
	Not useful	→								Very useful

VI-2. How useful do you think such a transformation would be to the field of public health in general?
Please circle the appropriate number, with 1 being "Not at all useful", and 10 being "Very useful"

0	1	2	3	4	5	6	7	8	9	10
	Not useful	→								Very useful

VI-3. Imagine you are a data custodian, and that a method has been developed to take your individual level data and mask it or change it somehow, while still keeping it at an individual-by-individual level. Would you allow such a method to be conducted on your data so that it can be shared with other public health professionals for public health research and practice?

☐ YES ☐ NO → Please explain why not ☐ MAYBE → Please explain

A specific disease or health condition will be used to test and evaluate the developed method(s). This condition must have a known aetiology, with well-known patterns and relationships, to serve as a starting point for the research. It must also be a disease of interest to the public health community.

VI-4. What diseases, health conditions, or databases most immediately come to mind as potential subjects for this research?

VI-5. Based on your knowledge of the condition you identified in the previous question, what relationships to the physical environment would a *transformation* as defined in the opening paragraph of this section have to retain in order for the data to be meaningful and useful to you (e.g. where patients or cases are relative to each other, to schools, to restaurants, etc; where schools are relative to a type of industry; etc…)?

Another proposed solution to the issue at hand is to build what are called automated *software agents*. You can think of these as applications that would go into a dataset wherever it is housed (i.e. at the custodian's location), perform the analyses for you (on the *personally identifiable data*) and return only the aggregated, and therefore anonymised, results. In other words, you would never see the actual data, but would have this "agent" do the analyses for you, directly on the *PID*; you simply get the results of the analyses, as long as, of course, they don't compromise privacy. As a simple analogy, it would be like you giving me an equation or function to perform on my data, and I giving you back the result of that function without you ever needing to see my actual data. Assuming the data custodians allow such a *software agent* to analyse their data and make the results available to the public health professional community:

VI-6. How useful do you think such a *software agent* would be to you in your current role?
Please circle the appropriate number, with 1 being "Not at all useful", and 10 being "Very useful"

0	1	2	3	4	5	6	7	8	9	10
	Not useful	→								Very useful

VI-7. How useful do you think such a *software agent* would be to the field of public health in general?
Please circle the appropriate number, with 1 being "Not at all useful", and 10 being "Very useful"

0	1	2	3	4	5	6	7	8	9	10
	Not useful	→								Very useful

VI-8. If you were (or are) a data custodian, would you allow such a *software agent* to access your data, conduct the analyses, and return the results to the public health professional community for research and analysis?

☐ YES ☐ NO → Please explain why not ☐ MAYBE → Please explain

VI-9. To summarise, if a solution is found such that privacy is no longer an issue, which of the following would you prefer? (Please select only **one**)

☐ I would prefer to be able to work directly with the raw data, so I can access information on a case-by-case basis.

☐ I have no need to see the raw data, and would prefer to access information and results on an aggregate basis.

SECTION VII – Qualitative Component

(~ 5 minutes)

VII-1. How knowledgeable do you consider yourself on privacy and confidentiality issues / legislation?
Please circle the appropriate number, 1 being "Not at all knowledgeable", and 10 being "Expert"

1	2	3	4	5	6	7	8	9	10

Not knowledgeable → Expert

VII-2. How do you feel about the impact of privacy and confidentiality legislation – in particular the restrictions on access to personally identifiable data (e.g. The Privacy Act, The Personal Information Protection and Electronic Documents Act, etc) – on public health?

VII-3. What do you think of the proposed research (development of a *transformation*)?

VII-4. What do you think of the "*software agent*" idea?

VII-5. Do you have any other thoughts or comments regarding this issue, the proposed research, or this questionnaire that you would like to share?

SECTION VIII – Further Participation and Contact

Please indicate your desired level of anonymity and interest in further participation; you may check multiple boxes as applicable. Please note that leaving this section empty will default your response to “absolute anonymity”, rendering you answers on this questionnaire personally unidentifiable and removing yourself from any further contact or participation.

☐ You may link my identity to my responses on this questionnaire for clarifications and follow-up
☐ Please send me a summary of the findings of this questionnaire, once completed
☐ Please send me periodic updates on the progress of this research by email to the address given below
☐ I am interested in piloting the results of this research

If you checked any of the above boxes, please complete your details below. Your personal information will be stored in a password-protected directory within the Public Health Agency of Canada, and will be protected according to the Access to Information Act and the Privacy Act.

Name: ____

Title: ____

Organization: ____

Address: ____

____

E-Mail: ____

Phone: ____

Preferred Method of Contact: ☐ Phone ☐ Fax ☐ E-Mail ☐ Mail

Are you in possession of any personally identifiable data that you can use for testing and evaluation of the developed transformation?

☐ YES ☐ NO

Dear Public Health Professional,

Thank you so much for taking the time to complete this questionnaire; your responses will help assess the impact of privacy and confidentiality legislation on public health research, and will be used to investigate and develop a disease-specific solution, which will, in turn, enhance strategic decisions and research in public health.

Once again, many thanks for your time, and I look forward to enhancing public health practice by exploring this issue, and its solution, further with you. Should you have any comments, questions or concerns, please feel free to send me an email or give me a ring.

Best wishes,

Philip AbdelMalik
