# Supplementary material for: The perceived impact of location privacy: A web-based survey of public health perspectives and requirements in the UK and Canada
Source: BMC Public Health. 2008 May 9;8:156. doi: 10.1186/1471-2458-8-156 (PMC2396622; doi:10.1186/1471-2458-8-156)
Supplement: Additional file 2 — L'impact de la protection des renseignements personnels sur la pratique en santé publique, CANADA. This is the paper version of the Canadian-French survey that was adapted for the web to collect public health professional perspectives and requirements on location-privacy in Canada. [file 1471-2458-8-156-S2.pdf]

L’impact de la protection des renseignements personnels sur la pratique en santé publique
CANADA

TEMPS REQUIS ESTIMÉ POUR RÉPONDRE À CE QUESTIONNAIRE : 20 À 30 MINUTES

Merci de prendre le temps de remplir ce questionnaire - votre contribution est bien appréciée et critique à la recherche.

Ce questionnaire est divisé en huit courtes sections pour mieux classer par catégorie et organiser les types d'information. Vous pouvez sauter pardessus n'importe quel question. Notez toutefois que chaque saut compromet la qualité de la recherche. Veuillez noter également que, à moins que vous choisissiez d'accomplir la section VIII du questionnaire, vos réponses demeureront anonymes. Assurez-vous svp que toutes vos réponses sont inscrites clairement.

Veuillez noter que vos réponses deviendront la propriété du gouvernement Canada, et comme tels, peuvent également être rendues disponibles pour être employées par l'agence de santé publique du Canada pour améliorer ses opérations et pour assurer des services à la communauté de santé publique. Ce questionnaire est mené par le bureau de la pratique en santé publique de l’agence de la santé publique du Canada. Donc, toutes les données rassemblées, y compris votre information personnelle, seront protégées selon la loi d'accès à l'information et la loi de protection des renseignements personnels du Canada. Notez également que, à moins que vous choisissiez d'accomplir la section VIII du questionnaire, vos réponses demeureront anonymes. Par conséquent, assurez-vous svp que toutes les réponses sont clairement marquées.

SECTION I – Un aperçu à votre sujet ...

(~ 5 minutes)

I-1 A quel niveau se situe la portée de **l’essentiel** de vos activités en santé publique ?
(Si plus d’un choix s’applique, veuillez ne cocher que la case correspondant au niveau **principal** de vos activités)

☐ International ☐ Amérique du Nord ☐ National ☐ Provincial/ Territorial ☐ Régional/ Local

I-2 Auprès de quel type d’organisation de santé publique travaillez-vous ou êtes-vous affilié ?
(Si plus d’un choix s’applique, veuillez ne cocher que la case correspondant au type d’organisation **principal** de vos activités)

☐ Agence de santé publique du Canada
☐ Santé Canada
☐ Autre agence gouvernementale fédérale
☐ Gouvernement provincial (ministère de la santé, etc)
☐ Autorité ou régie régionale ou locale
☐ Association Canadienne de santé publique
☐ Autre organisme non gouvernemental (provincial, épidémiologie, diabète, cancer, etc.)
☐ Université ou établissement d’enseignement
☐ Autre
(préciser svp) ____

I-3. Veuillez préciser le ou les domaine(s) d’expertise dans lesquels vous exercez présentement :
(Cocher toutes les mentions pertinentes)

☐ Santé des Autochtones
☐ Maladies chroniques (cancer, diabète, etc.)
☐ Santé publique des enfants / pédiatrique
☐ Maladies transmissibles / infectieuses
☐ Santé publique dentaire
☐ Protection civile / mesures d’urgence
☐ Environnement (pollution, climat, sécurité de l’eau et des aliments, etc.)
☐ Éthique, droit de la santé publique, protection des renseignements personnels, etc.
☐ Aliments et nutrition
☐ Génétique
☐ Services et soins de santé (besoins, prestation des services, etc.)
☐ Préjudices corporels / Invalidité
☐ Santé mentale et toxicomanies
☐ Santé en milieu de travail
☐ Déterminants sociaux de la santé (pauvreté, éducation, exclusion sociale, etc.)
☐ Surveillance
☐ Autre
(préciser svp) ____

I-4. Quelle attribution, parmi celles mentionnées ci-après, décrit le mieux votre rôle ou vos fonctions à titre de professionnel de la santé publique ?
(Si plus d’un choix s’applique, veuillez ne cocher que la case correspondant a votre rôle ou vos fonctions **principales**)

- ☐ Décideur / concepteur de politiques stratégiques
- ☐ Gestionnaire ou coordonnateur
- ☐ Consultant
- ☐ Autre (préciser svp) ____________________
- ☐ Recherche et analyse
- ☐ Intervenant de première ligne / soins aux patients / services cliniques

I-5. Dans le cadre de vos activités régulières, combien de temps consacrez-vous (approximativement, en pourcentage) ordinairement à l’accomplissent de chacune des tâches suivantes ?

Décideur / concepteur de politiques stratégiques	______%
Gestionnaire ou coordonnateur	______%
Consultant	______%
Recherche et analyse	______%
Intervenant de première ligne / soins aux patients / services cliniques	______%
Autre (comme indiqué dedans I-4)	______%

I-6. Dans l’accomplissement de quelle(s) tâche(s) mentionnées ci-dessus est-il **le plus probable** que vous utilisiez ou auriez besoin d’utiliser des renseignements nominatifs (permettant d’identifier un individu) ?

- ☐ Décideur / concepteur de politiques stratégiques
- ☐ Gestionnaire ou coordonnateur
- ☐ Consultant
- ☐ Autre (préciser svp) ____________________
- ☐ Recherche et analyse
- ☐ Intervenant de première ligne / soins aux patients / services cliniques

I-7 Avez-vous besoin ou prévoyez-vous le besoin d’inclure des données sur la zone géographique des données recueillies en matière de santé, dans le cadre de vos activités ou de celles de votre organisation ?

☐ OUI ☐ NON

I-8 Un système d’information géographique (SIG) est un outil vous permettant de visualiser et d’analyser vos données en y intégrant une composante spatiale, c’est-à-dire en tenant compte de leur emplacement géographique sur la planète. Dans l’accomplissement de quelle(s) tâche(s) le recours au SIG pourrait-il vous être utile ?

- ☐ Décideur / concepteur de politiques stratégiques
- ☐ Recherche et analyse
- ☐ Gestionnaire ou coordonnateur
- ☐ Intervenant de première ligne / soins aux patients / services cliniques
- ☐ Consultant
- ☐ Autre
  (préciser svp) ____

I-9 Quelle(s) application(s) de SIG utilisez-vous présentement ou avez-vous utilisées par le passé ?

- ☐ Générateur de cartes en santé publique (GCSP)
- ☐ Autre (disponibles sur le web; svp indiquez) ______________________________

Produits SIG utilisés en bureautique :

- ☐ ESRI (ArcGIS)
- ☐ MapInfo
- ☐ AutoDesk
- ☐ PCI Geomatics
- ☐ Intergraph
- ☐ Autre
  (préciser svp) ____

- ☐ Je n’ai jamais utilisé des applications de SIG, et je n’y vois aucune utilité dans mes activités
- ☐ Je n’ai jamais utilisé des applications de SIG, mais je souhaite en apprendre davantage à ce sujet

I-10 À quel niveau de géographie est-ce que vous visualisez vos données ou en faites une analyse spatiale pour chacun des produits que vous utilisez, le cas échéant ?

	GCSP	Autres niveaux disponibles sur le web	Produits SIG utilisés en bureautique
Latitude et Longitude	☐	☐	☐
Adresse civique	☐	☐	☐
Aire de diffusion	☐	☐	☐
Code postal	☐	☐	☐
Subdivision de recensement	☐	☐	☐
Division de recensement	☐	☐	☐
Région de tri d'acheminement	☐	☐	☐
Urbain – Rural	☐	☐	☐
Provincial	☐	☐	☐

Autre

(préciser svp) ____

I-11 Êtes-vous restreint dans l'utilisation de SIG ou devez-vous restreindre votre utilisation de SIG pour accomplir quelque activité de santé publique en raison de préoccupations au niveau de la protection des renseignements personnels (par exemple, de crainte que la carte ou les données soient susceptibles de permettre l'identification d'un individu ou d'une communauté) ?

☐ OUI → svp expliquez ☐ NON → svp expliquez

I-12 En faisant abstraction pour l'instant de vos préoccupations au niveau de la protection des renseignements personnels et selon votre réponse à la question précédente, à quel niveau de géographie est-ce que vous souhaiteriez ***idéalement*** pouvoir visualiser vos données ou en faire une analyse spatiale pour chacun des produits que vous utilisez, le cas échéant ?

	GCSP	Autres niveaux disponibles sur le web	Produits SIG utilisés en bureautique
Latitude et Longitude	☐	☐	☐
Adresse civique	☐	☐	☐
Aire de diffusion	☐	☐	☐
Code postal	☐	☐	☐
Urbain – Rural	☐	☐	☐

Autre

(préciser svp) ____

SECTION II – Accès actuel aux données

(~ 4 minutes)

Toutes les questions dans cette section concernent le rôle que vous avez identifié à la question I-6 dans la section I. Si vous n'avez pas accès à des données personnellement identifiables (PID) énumérées à la question II-1, cochez svp la dernière option à la question II-1 et sautez à la section III - Aucun accès courant aux données. Sinon, remplissez svp cette section, et passez ensuite à la section IV sur la protection des renseignements personnels.

REMARQUE : Dans ce questionnaire, le terme *accès* se rapporte à votre capacité *d'acquérir* effectivement des renseignements nominatifs de manière à pouvoir travailler directement avec ces données.

II-1. En tenant compte des tâches dans lesquelles vous êtes le plus susceptible d'avoir besoin d'utiliser des données nominatives, à quels renseignements nominatifs avez-vous présentement accès ? (Cocher toutes les mentions pertinentes)

- ☐ Prénom
- ☐ Nom
- ☐ Initiales
- ☐ Sexe
- ☐ Date de naissance/ Age
- ☐ Date de décès
- ☐ Numéro d'assurance-maladie
- ☐ Numéro d'identification d'une carte d'hôpital
- ☐ Médecin généraliste ou en médecine familiale
- ☐ Adresse civique
- ☐ Code postal
- ☐ Nom de la communauté
- ☐ Ville / Municipalité / Village
- ☐ Région / Zone géographique
- ☐ Latitude et Longitude

- ☐ Autre
  (préciser svp) ____

- ☐ **Je n'ai PAS accès présentement à l'un ou l'autre de ces éléments (svp sautez à la Section III)**

Les questions qui suivent se rapportent toutes aux renseignements nominatifs auxquels vous avez accès, tel que vous l'avez indiqué à la question précédente à ce sujet. Dans l'échelle des choix de réponse, encercler « 0 » si votre réponse est « Ne sais pas ».

II-2 Dans une perspective de protection des renseignements personnels et organisationnels, comment jugez-vous le degré de facilité avec lequel vous avez accès à des renseignements nominatifs lorsque vous en avez besoin ?
Encercler le chiffre correspondant à votre réponse, 1 = « Extrêmement difficile » et 10 = « Très facile »

Extrêmement difficile → Très facile

	Ne sais pas	1	2	3	4	5	6	7	8	9	10
Prénom	○	○	○	○	○	○	○	○	○	○	○
Nom	○	○	○	○	○	○	○	○	○	○	○
Initiales	○	○	○	○	○	○	○	○	○	○	○
Sexe	○	○	○	○	○	○	○	○	○	○	○
Date de naissance/ Age	○	○	○	○	○	○	○	○	○	○	○
Date de décès	○	○	○	○	○	○	○	○	○	○	○
Numéro d’assurance-maladie	○	○	○	○	○	○	○	○	○	○	○
Numéro d’identification d’une carte d’hôpital	○	○	○	○	○	○	○	○	○	○	○
Médecin généraliste ou en médecine familiale	○	○	○	○	○	○	○	○	○	○	○
Adresse civique	○	○	○	○	○	○	○	○	○	○	○
Code postal	○	○	○	○	○	○	○	○	○	○	○
Nom de la communauté	○	○	○	○	○	○	○	○	○	○	○
Ville / Municipalité / Village	○	○	○	○	○	○	○	○	○	○	○
Région / Zone géographique	○	○	○	○	○	○	○	○	○	○	○
Latitude / Longitude	○	○	○	○	○	○	○	○	○	○	○

II-3. En général, combien de fois accédez-vous aux renseignements nominatifs que vous avez mentionnés ci-dessus ?
Encercler le chiffre correspondant à votre réponse, 1 = « rarement » et 10 = « Tout le temps »

Rarement ——————————————► Tout le temps

	Non applicable	1	2	3	4	5	6	7	8	9	10
Prénom	○	○	○	○	○	○	○	○	○	○	○
Nom	○	○	○	○	○	○	○	○	○	○	○
Initiales	○	○	○	○	○	○	○	○	○	○	○
Sexe	○	○	○	○	○	○	○	○	○	○	○
Date de naissance/ Age	○	○	○	○	○	○	○	○	○	○	○
Date de décès	○	○	○	○	○	○	○	○	○	○	○
Numéro d’assurance-maladie	○	○	○	○	○	○	○	○	○	○	○
Numéro d’identification d’une carte d’hôpital	○	○	○	○	○	○	○	○	○	○	○
Médecin généraliste ou en médecine familiale	○	○	○	○	○	○	○	○	○	○	○
Adresse civique	○	○	○	○	○	○	○	○	○	○	○
Code postal	○	○	○	○	○	○	○	○	○	○	○
Nom de la communauté	○	○	○	○	○	○	○	○	○	○	○
Ville / Municipalité / Village	○	○	○	○	○	○	○	○	○	○	○
Région / Zone géographique	○	○	○	○	○	○	○	○	○	○	○
Latitude / Longitude	○	○	○	○	○	○	○	○	○	○	○

II-4. Dans quelle mesure ces renseignements nominatifs vous sont-ils utiles ou utiles dans l’accomplissement des rôles et responsabilités qui vous incombent ?
Encercler le chiffre correspondant à votre réponse, 1 = « Pas du tout utiles » et 10 = « Essentiels à l’accomplissement des rôles et responsabilités qui m’incombent »

Pas du tout utiles → Essentiels

	Ne sais pas	1	2	3	4	5	6	7	8	9	10
Prénom	○	○	○	○	○	○	○	○	○	○	○
Nom	○	○	○	○	○	○	○	○	○	○	○
Initiales	○	○	○	○	○	○	○	○	○	○	○
Sexe	○	○	○	○	○	○	○	○	○	○	○
Date de naissance/ Age	○	○	○	○	○	○	○	○	○	○	○
Date de décès	○	○	○	○	○	○	○	○	○	○	○
Numéro d’assurance-maladie	○	○	○	○	○	○	○	○	○	○	○
Numéro d’identification d’une carte d’hôpital	○	○	○	○	○	○	○	○	○	○	○
Médecin généraliste ou en médecine familiale	○	○	○	○	○	○	○	○	○	○	○
Adresse civique	○	○	○	○	○	○	○	○	○	○	○
Code postal	○	○	○	○	○	○	○	○	○	○	○
Nom de la communauté	○	○	○	○	○	○	○	○	○	○	○
Ville / Municipalité / Village	○	○	○	○	○	○	○	○	○	○	○
Région / Zone géographique	○	○	○	○	○	○	○	○	○	○	○
Latitude / Longitude	○	○	○	○	○	○	○	○	○	○	○

II-5. Quel impact aurait le fait de perdre votre accès à ces renseignements nominatifs sur la qualité de votre travail et les décisions en matière de santé publique qui en résultent ?
Encercler le chiffre correspondant à votre réponse, 1 = « Aucun impact – aucune incidence sur la qualité » et 10 = « Impact sérieux – les résultats et les décisions en seraient gravement compromis »

Aucun impact → Impact sérieux

	Ne sais pas	1	2	3	4	5	6	7	8	9	10
Prénom	○	○	○	○	○	○	○	○	○	○	○
Nom	○	○	○	○	○	○	○	○	○	○	○
Initiales	○	○	○	○	○	○	○	○	○	○	○
Sexe	○	○	○	○	○	○	○	○	○	○	○
Date de naissance/ Age	○	○	○	○	○	○	○	○	○	○	○
Date de décès	○	○	○	○	○	○	○	○	○	○	○
Numéro d’assurance-maladie	○	○	○	○	○	○	○	○	○	○	○
Numéro d’identification d’une carte d’hôpital	○	○	○	○	○	○	○	○	○	○	○
Médecin généraliste ou en médecine familiale	○	○	○	○	○	○	○	○	○	○	○
Adresse civique	○	○	○	○	○	○	○	○	○	○	○
Code postal	○	○	○	○	○	○	○	○	○	○	○
Nom de la communauté	○	○	○	○	○	○	○	○	○	○	○
Ville / Municipalité / Village	○	○	○	○	○	○	○	○	○	○	○
Région / Zone géographique	○	○	○	○	○	○	○	○	○	○	○
Latitude / Longitude	○	○	○	○	○	○	○	○	○	○	○

II-6. À quels renseignements nominatifs n’avez-vous **PAS** présentement accès, mais dont l’accès vous serait bénéfique à votre avis à fin d’améliorer votre travail et les décisions en matière de santé publique qui en résultent ? (Cocher toutes les mentions pertinentes)

- ☐ Prénom
- ☐ Nom
- ☐ Initiales
- ☐ Sexe
- ☐ Date de naissance/ Age
- ☐ Date de décès
- ☐ Numéro d’assurance-maladie
- ☐ Numéro d’identification d’une carte d’hôpital
- ☐ Médecin généraliste ou en médecine familiale
- ☐ Adresse civique
- ☐ Code postal
- ☐ Nom de la communauté
- ☐ Ville / Municipalité / Village
- ☐ Région / Zone géographique
- ☐ Latitude et Longitude

- ☐ Autre
  (préciser svp) ____

- ☐ Aucun

PERSON SHOULD NOW BE TAKEN TO SECTION IV – PRIVACY ISSUES

SECTION III – Absence d’accès aux données

(~ 2 minutes)

If the person completed Section II above, then skip to Section IV – Privacy Issues.

Dans l’échelle des choix de réponse, encercler « 0 » si votre réponse est « Ne sais pas ».

REMARQUE : Dans ce questionnaire, le terme *accès* se rapporte à votre capacité *d’acquérir* effectivement des renseignements nominatifs de manière à pouvoir travailler directement avec ces données.

III-1. L’accès à quels renseignements nominatifs, parmi la liste suivante, vous serait bénéfique à votre avis à fin d’améliorer votre travail et les décisions en matière de santé publique qui en résultent ? (Cocher toutes les mentions pertinentes)

- ☐ Prénom
- ☐ Nom
- ☐ Initiales
- ☐ Sexe
- ☐ Date de naissance/ Age
- ☐ Date de décès
- ☐ Numéro d’assurance-maladie
- ☐ Numéro d’identification d’une carte d’hôpital
- ☐ Médecin généraliste ou en médecine familiale
- ☐ Adresse civique
- ☐ Code postal
- ☐ Nom de la communauté
- ☐ Ville / Municipalité / Village
- ☐ Région / Zone géographique
- ☐ Latitude et Longitude

- ☐ Autre
  (préciser svp) ____

- ☐ Aucun

III-2. Dans quelle mesure l’accès aux renseignements nominatifs que vous avez indiqués ci-dessus serait-il utile à l’accomplissement des rôles et responsabilités qui vous incombent (que vous avez indiqués en réponse à la Section I) ?
Encercler le chiffre correspondant à votre réponse, 1 = « Pas du tout utile » et 10 = « Très utile – aideraient grandement les rôles et responsabilités qui m’incombent »

Pas du tout utile → Très utile

	Ne sais pas	1	2	3	4	5	6	7	8	9	10
Prénom	○	○	○	○	○	○	○	○	○	○	○
Nom	○	○	○	○	○	○	○	○	○	○	○
Initiales	○	○	○	○	○	○	○	○	○	○	○
Sexe	○	○	○	○	○	○	○	○	○	○	○
Date de naissance/ Age	○	○	○	○	○	○	○	○	○	○	○
Date de décès	○	○	○	○	○	○	○	○	○	○	○
Numéro d’assurance-maladie	○	○	○	○	○	○	○	○	○	○	○
Numéro d’identification d’une carte d’hôpital	○	○	○	○	○	○	○	○	○	○	○
Médecin généraliste ou en médecine familiale	○	○	○	○	○	○	○	○	○	○	○
Adresse civique	○	○	○	○	○	○	○	○	○	○	○
Code postal	○	○	○	○	○	○	○	○	○	○	○
Nom de la communauté	○	○	○	○	○	○	○	○	○	○	○
Ville / Municipalité / Village	○	○	○	○	○	○	○	○	○	○	○
Région / Zone géographique	○	○	○	○	○	○	○	○	○	○	○
Latitude / Longitude	○	○	○	○	○	○	○	○	○	○	○

III-3 Dans quelle mesure cela serait-il facile pour vous d’accéder aux renseignements nominatifs que vous avez identifiés ci-dessus, si vous deviez en avoir besoin ?

Encercler le chiffre correspondant à votre réponse, 1 = « Impossible » et 10 = « Très facile »

Impossible → Très facile

	Ne sais pas	1	2	3	4	5	6	7	8	9	10
Prénom											
Nom											
Initiales											
Sexe											
Date de naissance/ Age											
Date de décès											
Numéro d’assurance-maladie											
Numéro d’identification d’une carte d’hôpital											
Médecin généraliste ou en médecine familiale											
Adresse civique											
Code postal											
Nom de la communauté											
Ville / Municipalité / Village											
Région / Zone géographique											
Latitude / Longitude											

III-4. Quel impact le fait de ne pas avoir accès à ces renseignements nominatifs a-t-il sur la qualité de votre travail et les décisions en matière de santé publique qui en résultent ?

Encercler le chiffre correspondant à votre réponse, 1 = « Aucun impact – aucune incidence sur la qualité » et 10 = « Impact sérieux – les résultats et les décisions ont été gravement compromis »

Aucun Impact ⟶ Impact sérieux

	Ne sais pas	1	2	3	4	5	6	7	8	9	10
Prénom	○	○	○	○	○	○	○	○	○	○	○
Nom	○	○	○	○	○	○	○	○	○	○	○
Initiales	○	○	○	○	○	○	○	○	○	○	○
Sexe	○	○	○	○	○	○	○	○	○	○	○
Date de naissance/ Age	○	○	○	○	○	○	○	○	○	○	○
Date de décès	○	○	○	○	○	○	○	○	○	○	○
Numéro d’assurance-maladie	○	○	○	○	○	○	○	○	○	○	○
Numéro d’identification d’une carte d’hôpital	○	○	○	○	○	○	○	○	○	○	○
Médecin généraliste ou en médecine familiale	○	○	○	○	○	○	○	○	○	○	○
Adresse civique	○	○	○	○	○	○	○	○	○	○	○
Code postal	○	○	○	○	○	○	○	○	○	○	○
Nom de la communauté	○	○	○	○	○	○	○	○	○	○	○
Ville / Municipalité / Village	○	○	○	○	○	○	○	○	○	○	○
Région / Zone géographique	○	○	○	○	○	○	○	○	○	○	○
Latitude / Longitude	○	○	○	○	○	○	○	○	○	○	○

SECTION IV – Protection des renseignements personnels

(~ 3 minutes)

Cette section a trait au domaine de la santé publique dans son ensemble; l'emploi de l'expression « pratique en santé publique » vise les diverses activités réalisées dans ce domaine : recherche, surveillance de la santé, prestation des services de santé, élaboration des politiques et prise de décisions stratégiques, etc. L'objectif est d'obtenir votre opinion, en tant que professionnel de la santé publique, au sujet de l'impact global de la restriction de l'accès à des renseignements nominatifs *sur la pratique en santé publique* au Canada. Ces questions ont pour objet de recueillir votre opinion à ce sujet; si vous n'êtes pas certain de la réponse à donner à une question donnée dans cette section, veuillez quand même donner une réponse selon votre bon jugement.

REMARQUE : **Dans ce questionnaire, le terme *accès* se rapporte à votre capacité *d'acquérir* effectivement des renseignements nominatifs de manière à pouvoir travailler directement avec ces données.**

IV-1. À votre avis, les restrictions actuelles sur l'accès aux renseignements nominatifs constituent-elles un obstacle à quelque volet de la *pratique en santé publique* (recherche, surveillance, etc.) ?
Encercler le chiffre correspondant à votre réponse, 1 = « Pas du tout un obstacle » et 10 = « Elles constituent une grave menace à l'exercice efficace et convenable de la pratique en santé publique »

1	2	3	4	5	6	7	8	9	10

Pas du tout un obstacle → Une grave menace

IV-2. Dans quelle mesure seriez-**vous** disposé à ce que d'autres professionnels du domaine de la santé publique aient accès à des renseignements nominatifs **vous concernant** aux fins de recherche et d'analyse en santé publique (**votre** adresse, date de naissance, etc.) à fin d'améliorer la prestation des services en matière de santé publique, etc. ?

☐ D'accord, pas de problème ☐ Aucunement ☐ Pas certain → Préciser pourquoi :

IV-3a. À votre avis, dans quelle proportion le grand public est-il au courant de l'impact de l'accès restreint aux renseignements nominatifs sur la pratique en santé publique ?
Svp encercler la proportion approximative

10%	20%	30%	40%	50%	60%	70%	80%	90%	100%

Personne → Tout le monde

IV-3b. De quelle manière croyez-vous que l'on pourrait augmenter cette proportion ?

IV-4a. À votre avis, quelle proportion du grand public permettrait que l'on utilise les renseignements nominatifs des individus aux fins de la pratique en santé publique si on interrogeait les gens et les sensibilisait au sujet de l'utilité de tels renseignements aux fins de la pratique en santé publique ?
Svp encercler la proportion approximative

10%	20%	30%	40%	50%	60%	70%	80%	90%	100%

Personne → Tout le monde

IV-4b. Comment pourrait-on augmenter cette proportion ?

SECTION V – Données actuelles et dispositions pour l'obtention d'autres renseignements ... (~ 2 minutes)

Cette section vise à recueillir des informations au sujet du partage des renseignements nominatifs au sein de votre organisation et entre des organisations.
Dans l'échelle des choix de réponse, encercler « 0 » si votre réponse est « Ne sais pas ».

V-1. À votre avis, quel serait le **principal** obstacle au partage ou à l'acquisition de renseignements nominatifs liés à des informations sur la santé des individus ?
(Veuillez ne cocher qu'un seul choix – il s'agit de faire valoir votre opinion !)

☐ Les lois nationales ☐ La réprobation du public ☐ La bureaucratie
☐ L'absence de connaissances ☐ La paranoïa du public ☐ La paranoïa des praticiens

☐ Autre (préciser svp) ____

V-2. Est-ce que vous ou votre organisation recueillez à l'heure actuelle des renseignements nominatifs en matière de santé à quelque fin que ce soit (recherche, surveillance, prestation des services, etc.), ou agissez à titre de dépositaire de tels renseignements ?

☐ OUI ☐ NON **Go to Section VI**

V-3. À quelles fins spécifiques ces renseignements sont-ils recueillis ? (Cocher toutes les mentions pertinentes)

☐ Recherche ☐ Surveillance ☐ Prestation des services
☐ Autre
(préciser svp) ____

V-4. Quels renseignements sont recueillis ?

☐ Prénom
☐ Nom
☐ Initiales
☐ Sexe
☐ Date de naissance/ Age
☐ Date de décès
☐ Numéro d'assurance-maladie
☐ Numéro d'identification d'une carte d'hôpital
☐ Médecin généraliste ou en médecine familiale

☐ Adresse civique
☐ Code postal
☐ Nom de la communauté
☐ Ville / Municipalité / Village
☐ Région / Zone géographique
☐ Latitude et Longitude

☐ Autre
(préciser svp) ____

V-5. Dans quelle mesure est-ce difficile pour les professionnels de la santé publique, dont vous-même, d'avoir accès aux renseignements nominatifs dont vous disposez et autres données sur la santé qui y sont liées lorsque ces renseignements ou données sont situées à l'extérieur du périmètre immédiat de votre équipe de travail, mais à l'intérieur …

Impossible → Très facile

	Ne sais pas	1	2	3	4	5	6	7	8	9	10
De votre organisation ?	○	○	○	○	○	○	○	○	○	○	○
Du gouvernement fédéral	○	○	○	○	○	○	○	○	○	○	○
De votre gouvernement provincial	○	○	○	○	○	○	○	○	○	○	○
D'un gouvernement provincial autre que le vôtre ?	○	○	○	○	○	○	○	○	○	○	○
D'une régie régionale ou autre autorité chargée de la santé publique ?	○	○	○	○	○	○	○	○	○	○	○
D'une université ou établissement de recherche ?	○	○	○	○	○	○	○	○	○	○	○
De l'administration d'un gouvernement étranger (p. ex. le CDC aux États-Unis, le NHS au Royaume-Uni, etc.)	○	○	○	○	○	○	○	○	○	○	○
De l'organisation de santé mondiale	○	○	○	○	○	○	○	○	○	○	○

SECTION VI – Solutions et recherche

(~ 7 minutes)

La recherche proposée s’intéressera à l’application d’une méthode (que l’on appelle *transformation*) à des données relatives à la santé publique de manière à ce que les liens importants entre les données et parmi celles-ci soient préservés, tout en rendant anonyme l’identité de l’individu. Par exemple, si l’on étudie le phénomène d’une éclosion d’une maladie infectieuse chez les enfants, vous vous intéresseriez à savoir où sont situés les enfants infectés les uns par rapport aux autres, ainsi qu’où sont situés les écoles, arénas, centres communautaires, etc.. Vous pourriez alors préserver *les liens* entre ces points d’intérêt et changer tous les autres éléments, de manière à ce que les points d’intérêt ainsi ciblés ne puissent être retracés vers l’identité des individus en jeu. Ainsi, vous avez réussi à *transformer* les données de telle sorte que les données soumises à l’analyse soient visibles au niveau individuel comme tel, mais sans pouvoir établir précisément à quelle personne elles se rapportent (les renseignements deviennent alors anonymes). Ceci suppose que les dépositaires des renseignements et des données permettent que les données issues de cette *transformation* soient mises à la disposition des professionnels de la santé publique .
Dans l’échelle des choix de réponse, encercler « 0 » si votre réponse est « Ne sais pas ».

VI-1. Dans quelle mesure une telle transformation vous serait-elle utile dans vos fonctions actuelles ?
Encercler le chiffre correspondant à votre réponse, 1 = « Pas du tout utile » et 10 = « Très utile – aideraient grandement les rôles et responsabilités qui m’incombent »

0	1	2	3	4	5	6	7	8	9	10
	Pas du tout utile									Très utile

VI-2. Dans quelle mesure estimez-vous qu’une telle transformation pourrait être utile au domaine de la santé publique en général ?
Encercler le chiffre correspondant à votre réponse, 1 = « Pas du tout utile » et 10 = « Très utile – aideraient grandement les rôles et responsabilités qui m’incombent »

0	1	2	3	4	5	6	7	8	9	10
	Pas du tout utile									Très utile

VI-3. Imaginez que vous êtes un dépositaire de données et qu’une méthode a été mise au point pour récupérer les renseignements nominatifs, les « masquer » ou les modifier d’une certaine façon, tout en les préservant au niveau individuel, individu par individu. Est-ce que vous autoriseriez que l’on utilise une telle méthode sur vos données, pour que le résultat puisse être partagé avec d’autres professionnels de la santé publique aux fins de la recherche et de la pratique en santé publique ?

☐ OUI ☐ NON → Préciser pourquoi : ☐ Peut-être → Préciser pourquoi :

Une maladie ou un état de santé spécifique serviront à mettre à l'essai et à évaluer la ou les méthodes élaborées. La maladie ou l'état de santé choisi devra posséder une étiologie connue, avec des modèles et des liens bien connus, à fin de servir comme point de départ à la recherche. La maladie ou l'état de santé choisi devra également être d'un intérêt certain pour la communauté de la santé publique.

VI-4. Quelle maladie, quel état de santé, ou quelle base de données vous viennent spontanément à l'esprit à titre de sujets potentiels aux fins de cette recherche ?

VI-5. Suivant vos connaissances au sujet de l'état de santé ou de la maladie que vous avez signalés dans votre réponse à la question précédente, quels liens à l'environnement physique une *transformation* (telle que définie au paragraphe introductif de la présente section) devra-t-elle conserver à fin que les données demeurent pertinentes et utiles à vos fins. Par exemple : Où se trouvent les patients ou les cas les uns par rapport aux autres, par rapport aux écoles, restaurants, etc.; où les écoles se trouvent par rapport à un type d'industrie, etc. ?

Une autre solution proposée à la problématique consiste à construire ce que l'on appelle des *agents logiciels* automatisés. Il s'agit d'applications qui s'insèrent dans l'ensemble de données visé, là où il est hébergé (i.e., à l'établissement du dépositaire des données), exécutent les analyses pour vous (*sur les renseignements nominatifs*) et vous retournent uniquement les résultats agrégés, lesquels sont ainsi rendus anonymes. En d'autres termes, vous ne verrez jamais les données comme tel, mais « l'agent » effectuera les analyses pour vous, directement sur les renseignements nominatifs, et vous obtenez uniquement les résultats de l'analyse, dans la mesure où ils ne compromettent pas la protection des renseignements personnels. Par analogie, c'est comme si vous me donniez une équation ou une fonction à exécuter avec les données que je possède, et que je vous remettrais ensuite le résultat de cette fonction sans que vous ayez besoin de voir mes données. En supposant que les dépositaires de données permettent qu'un tel *agent logiciel* analyse leurs données et rendent disponibles les résultats de l'analyse à la communauté des professionnels de la santé publique ...

VI-6. Dans quelle mesure un tel agent logiciel vous serait-il utile dans vos fonctions actuelles ? Encercler le chiffre correspondant à votre réponse, 1 = « Pas du tout utile » et 10 = « Très utile – aideraient grandement les rôles et responsabilités qui m'incombent »

0	1	2	3	4	5	6	7	8	9	10

Pas du tout utile → Très utile

VI-7. Dans quelle mesure estimez-vous qu'une tel agent logiciel pourrait être utile au domaine de la santé publique en général ?
Encercler le chiffre correspondant à votre réponse, 1 = « Pas du tout utile » et 10 = « Très utile – aideraient grandement les rôles et responsabilités qui m'incombent »

0	1	2	3	4	5	6	7	8	9	10
	Pas du tout utile									Très utile

VI-8. Si vous étiez (ou êtes) un dépositaire de données, est-ce que vous autoriseriez un tel agent logiciel à accéder à vos données, à procéder à leur analyse, et à en remettre les résultats à la communauté des professionnels de la santé publique à des fins d'analyse et de recherche ?

☐ OUI ☐ NON → Préciser pourquoi : ☐ Peut-être → Préciser pourquoi :

VI-9. En résumé, si une solution était trouvée de telle sorte que la protection des renseignements personnels ne serait plus problématique, quelle méthode est-ce que vous préféreriez utiliser ?
(Veuillez ne cocher qu'un seul choix)

☐ Je préfèrerais pouvoir travailler directement avec les données brutes, à fin de pouvoir accéder aux renseignements au cas par cas.

☐ Je n'ai pas besoin de voir les données brutes, et je préfèrerais alors avoir accès aux informations et aux résultats en leur forme agrégée.

SECTION VII – Aspects qualitatifs

(~ 5 minutes)

VII-1. Comment considérez-vous l'état de vos connaissances sur les questions et les lois se rapportant à la protection et la confidentialité des renseignements personnels ?
Encercler le chiffre correspondant à votre réponse, 1 = « Aucune connaissance particulière à ce sujet » et 10 = « Expert »

1	2	3	4	5	6	7	8	9	10

Aucune connaissance → Expert

VII-2. Que pensez-vous de l'impact des lois en matière de protection des renseignements personnels et de confidentialité sur la santé publique – en particulier en ce qui concerne les restrictions à l'accès aux renseignements nominatifs (*Loi sur la protection des renseignements personnels*, *Loi sur la protection des renseignements personnels et les documents électroniques*, etc.) ?

VII-3. Que pensez-vous de la recherche proposée (mise au point d'une méthode de *transformation*) ?

VII-4. Que pensez-vous de l'idée de l'*agent logiciel* ?

VII-5. Avez-vous des idées ou des commentaires à formuler au sujet de cette problématique, de la recherche proposée ou de ce questionnaire ?

SECTION VIII – Participation future et communications

Veuillez indiquer ici le degré d'anonymat que vous souhaitez et votre intérêt à participer à la présente initiative. Vous pouvez cocher plusieurs cases au besoin. Veuillez noter qu'en laissant la présente section en blanc, votre réponse sera considérée par défaut comme étant « anonymat absolu », rendant vos réponses au présent questionnaire non identifiables au plan individuel et vous soustrayant à toute communication ou participation future à ce sujet.

- ☐ Vous pouvez lier mon identité à mes réponses au présent questionnaire aux fins de clarification et de suivi
- ☐ Veuillez me transmettre un sommaire des résultats du présent questionnaire une fois l'analyse achevée
- ☐ Veuillez me transmettre des suivis périodiques sur la progression de cette recherche, par courriel à l'adresse électronique indiquée ci-dessous
- ☐ Je suis intéressé (e) à participer aux essais pilote des résultats de cette recherche

Veuillez indiquer vos coordonnées à l'endroit prévu ci-après. Vos renseignements personnels seront conservés dans un répertoire protégé par mot de passe au sein de l'Agence de santé publique du Canada, et seront protégés conformément aux dispositions de la *Loi sur l'accès à l'information* et de la *Loi sur la protection des renseignements personnels*.

Nom: ____________________

Titre: ____________________

Organisation: ____________________

Adresse: ____________________

____

Courriel: ____________________

Téléphone: ____________________

Méthode de communication préférée : ☐ Téléphone ☐ Télécopieur ☐ Courriel ☐ Par la poste

Êtes-vous en possession de renseignements nominatifs que vous pourriez utiliser aux fins des essais et de l'évaluation de la méthode de transformation mise au point dans le cadre de ce projet ?

☐ OUI ☐ NON

Cher(e) professionnel(le) de santé publique,

Grand merci d’avoir pris le temps de remplir ce questionnaire. Vos réponses aideront à évaluer l'impact de la législation de protection de renseignements personnels et de confidentialité sur la recherche en santé publique, et seront employées pour étudier et développer des solutions spécifiques à des maladies précises. En bout de ligne, vous nous aidez à augmenter la qualité des décisions stratégiques et de la recherche en la santé publique.

Encore une fois, je tiens à vous remercier et j’anticipe d’avoir l’occasion dans le futur d’explorer à fond ces questions avec vous et d’ainsi améliorer la pratique de la santé publique. N’hésitez pas à m’appeler ou à m’envoyer un courriel, pour toute question ou commentaire.

Bien à vous,

Philip AbdelMalik
